# Combining Radiology and Pathology for Automatic Glioma Classification

**DOI:** 10.3389/fbioe.2022.841958

**Published:** 2022-03-21

**Authors:** Xiyue Wang, Ruijie Wang, Sen Yang, Jun Zhang, Minghui Wang, Dexing Zhong, Jing Zhang, Xiao Han

**Affiliations:** ^1^ College of Biomedical Engineering, Sichuan University, Chengdu, China; ^2^ College of Computer Science, Sichuan University, Chengdu, China; ^3^ School of Automation Science and Engineering, Xi’an Jiaotong University, Xi’an, China; ^4^ Tencent AI Lab, Shenzhen, China; ^5^ Pazhou Lab, Guangzhou, China; ^6^ State Key Laboratory for Novel Software Technology, Nanjing University, Nanjing, China

**Keywords:** convolutional neural networks, deep learning, glioma, pathology, magnetic resonance image

## Abstract

Subtype classification is critical in the treatment of gliomas because different subtypes lead to different treatment options and postoperative care. Although many radiological- or histological-based glioma classification algorithms have been developed, most of them focus on single-modality data. In this paper, we propose an innovative two-stage model to classify gliomas into three subtypes (i.e., glioblastoma, oligodendroglioma, and astrocytoma) based on radiology and histology data. In the first stage, our model classifies each image as having glioblastoma or not. Based on the obtained non-glioblastoma images, the second stage aims to accurately distinguish astrocytoma and oligodendroglioma. The radiological images and histological images pass through the two-stage design with 3D and 2D models, respectively. Then, an ensemble classification network is designed to automatically integrate the features of the two modalities. We have verified our method by participating in the MICCAI 2020 CPM-RadPath Challenge and won 1st place. Our proposed model achieves high performance on the validation set with a balanced accuracy of 0.889, Cohen’s Kappa of 0.903, and an F1-score of 0.943. Our model could advance multimodal-based glioma research and provide assistance to pathologists and neurologists in diagnosing glioma subtypes. The code has been publicly available online at https://github.com/Xiyue-Wang/1st-in-MICCAI2020-CPM.

## 1 Introduction

Glioma is one of the most common tumors originating from the brain, which accounts for about 80% of malignant brain tumors in adults (Banerjee et al., 2020). It is characterized by high morbidity, high recurrence, high mortality, and low cure rate (Ostrom et al., 2018). Glioma can be divided into three subtypes (Louis et al., 2016), such as glioblastoma, oligodendroglioma, and astrocytoma. The timely detection and treatment for each glioma subtype can effectively reduce mortality. For patients, different glioma subtypes means different risks (Mesfin and Al-Dhahir, 2019; Wang et al., 2019; Ostrom et al., 2020). For neurologists, accurate classification of glioma subtypes is critical to help customize proper therapeutic intervention (Decuyper et al., 2018). Therefore, glioma subtype classification has important implications.

Before 2016, the classification of glioma subtypes relied mainly on purely histopathological criteria (Louis et al., 2007). In the 2016 report of the World Health Organization (WHO) on the classification of central nervous system (CNS) tumors, molecular parameters were used for the first time to diagnose CNS tumors. Isocitrate dehydrogenase genes mutation, 1p/19q codeletion, and histone H3 genes mutations became decisive markers for the classification of diffuse gliomas (Louis et al., 2016). However, the tools for molecular analysis of tumors are not readily available in areas with low medical resource settings. Thus, it leaves room for glioma diagnosis based only on histopathological analysis (Louis et al., 2016; Pei et al., 2021). Also, non-invasive radiology images (e.g., magnetic resonance imaging, MRI) can also offer an alternative for tumor classification (Reza et al., 2019).

**FIGURE 1:**
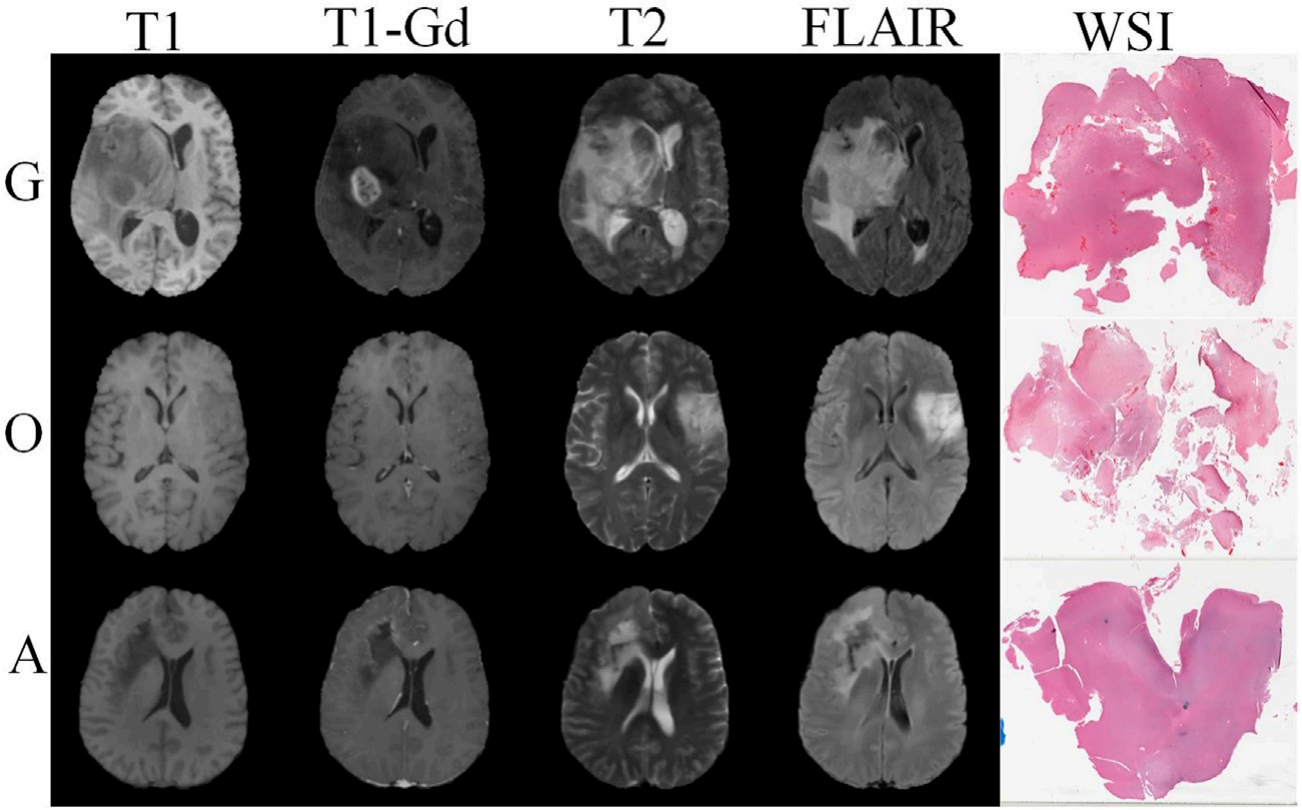
Visualization of glioblastoma (G), oligodendroglioma (O), and astrocytoma (A) in four sequences of MRI images and paired pathology images.

There are two common ways to observe gliomas (Mesfin and Al-Dhahir, 2019), as shown in Figure 1. MRI is a non-invasive technique, which provides images of the brain in 2D and 3D formats (Abdelaziz Ismael et al., 2020). Generally, there are four different MRI sequences, including the T1-weighted (T1), the T1-weighted gadolinium contrasted (T1-Gd), the T2-weighted (T2), and the T2-weighted fluid-attenuated inversion recovery (FLAIR). Different glioma subtypes have different radiological features. The use of radiological images alone may not be sufficient to reliably distinguish different glioma subtypes (van Lent et al., 2020). In addition to MRI, hematoxylin and eosin (H&E) stained tissue biopsy image is another technique to observe brain tumors. It can provide histological features (e.g. necrosis, hemorrhage, polymorphism, and nuclear heterogeneity, etc.) to distinguish glioma subtypes (Wesseling and Capper, 2018). The histopathological images are often considered as the gold standard for tumor diagnosis (Jothi and Rajam, 2017). However, it provides only histological information and is not comprehensive enough. Clinical studies have shown that using combined information from MRI scans and tissue biopsies is more helpful in the diagnosis of gliomas than using unimodal images (Young et al., 2015). However, the viewing of multimodal images is time-consuming and subjective. Even among experts, the diagnosis for the same tumor image (especially samples with complex feature information) is often inconsistent, which is called interobserver variability (Ker et al., 2019; Faust et al., 2019; van den Bent, 2010). With the increasing number of patients and the limited number of pathologists, this variability needs to be solved urgently (Ohgaki et al., 2004; Okamoto et al., 2004). Deep learning is a powerful tool that can not only provide physicians with more objective clinical references but also improve the efficiency in the tumor classification process (LeCun et al., 2015; Mobadersany et al., 2018; Faust et al., 2019). This is a possible alternative to using deep neural networks to learn different types of image features for glioma classification.

In this paper, we train a 3D fully convolutional network for radiology images classification (3D MRI model) and a 2D fully convolutional network for histopathological whole-slide image (WSI) classification (2D WSI model). In the experiments, it is easy to encounter the same problem as clinicians, where the features of glioblastoma are easy to learn while astrocytoma and oligodendroglioma are difficult to distinguish. This is due to the presence of “mixed gliomas” (Huse et al., 2015). Some gliomas contain a mixed feature of astrocytoma and oligodendroglioma, which makes the classification of astrocytoma and oligodendroglioma extremely challenging. To address this problem, we adopt a two-stage strategy that focuses on two different classification tasks, respectively. In the first stage of the learning task, the model classifies brain tumors into glioblastoma and others. In the second stage, the model focuses on learning the differences between oligodendroglioma and astrocytoma. Meanwhile, this approach alleviates the data imbalance problem to some extent. The MRI and WSI data pass through the two-stage design with their corresponding 3D and 2D models, respectively. Then, an ensemble classification network is designed to automatically integrate the features of the two modalities.

The contributions of our work are summarized as follows:

• We design two complementary MRI- and WSI-based models with ensemble learning to achieve higher diagnostic performance than most glioma grading methods.
• To address the clinical problem of differentiating mixed gliomas, we propose a two-stage strategy that allows the model to focus on learning mixed features between astrocytoma and oligodendroglioma with good performance.
• Our method achieves the best classification performance in the MICCAI 2020 CPM-RadPath Challenge. Our model and results can be used as benchmarks for automatic glioma classification algorithms. The code is publicly available for others to conduct reproducible research.

The rest of the paper is organized as follows: Section 2 shows the related work; Section 3 describes our algorithm implementation; Section 4 presents the data and experimental results; Section 5 summarizes our work.

## 2 Related Work

Deep learning has achieved remarkable success in the computer vision community (Badrinarayanan et al., 2017; Isola et al., 2017; Pelt and Sethian, 2018). Benefiting from its superior performance, deep learning has also been widely used in the field of medical image processing, such as prostate MRI analysis (Milletari et al., 2016; Rundo et al., 2019; Rundo et al., 2020), neuronal structure segmentation (Ronneberger et al., 2015), brain tumor detection (Han et al., 2019), etc. For computer-assisted brain glioma diagnosis, current methods are based on unimodal (MRI or WSI). There is still a lack of multimodality-based glioma classification studies. Next, we will review MRI- and WSI-based glioma classification methods, respectively.

### 2.1 MRI-Based Approaches

Many methods have been proposed to classify gliomas using MRI images through deep learning methods based on radiological characteristics (Mohan and Subashini, 2018).

#### 2.1.1 Single Sequence-Based Approaches

Some studies focus on the glioma classification with single sequence images, such as the T1 sequence images (Hsieh et al., 2017; Kaya et al., 2017; Ge et al., 2018; Yang et al., 2018; Abdelaziz Ismael et al., 2020), the T1-Gd sequence images (Koley et al., 2016; Singh et al., 2017; Jang et al., 2018; Dikici et al., 2020; Zhou et al., 2020), the T2 sequence images (Wu et al., 2017; Yogananda et al., 2020), and the FLAIR sequence images (Gao et al., 2016; Wu et al., 2018; Su et al., 2019). Single sequence image diagnosis often relies on limited radiological features (Arbizu et al., 2011).

#### 2.1.2 Multiple Sequence-Based Approaches

There are also some studies that classify tumors based on multiple sequences (T1 with T1-Gd, T1 with T2, and others) in MRI (Wu et al., 2019; Alis et al., 2020; Chen et al., 2020; Hamghalam et al., 2020; Lu et al., 2020; Zhang et al., 2020). However, most of these studies simply divide gliomas into high-grade gliomas (HGG) and low-grade gliomas (LGG). Further work on subtype classification is relatively scarce, which is due to the difficulty of subtype characterization using MRI (Reifenberger et al., 2017).

### 2.2 WSI-Based Approaches

Some other methods have also been proposed to automatically classify gliomas based on histological features by deep learning methods, which will greatly improve diagnostic efficiency and improve patient outcomes (Jothi and Rajam, 2017; Mobadersany et al., 2018; Jin et al., 2020).

#### 2.2.1 Glioma Binary Classification

Several studies possess excellent performance in the WSI-based binary glioma classification. Ertosun et al. first proposed a modular approach to apply convolutional neural network for histopathological glioma classification (Ertosun and Rubin, 2015). Then, Yonekura et al. further investigated the automated glioma analysis method with deep learning techniques (Yonekura et al., 2017). Rathore et al. distinguished gliomas by learning phenotypic information (Rathore et al., 2020). Hou et al. trained patch-level classifiers to accomplish the glioma classification (Hou et al., 2016). While Zhu et al. learned patient-specific information from WSI for classification (Zhu et al., 2017).

All these methods use The Cancer Genome Atlas (TCGA) public dataset. However, limited by image annotation, they also simply classified gliomas into glioblastoma (GBM) and LGG. This does not provide substantial help for better treatment. There are also some studies that have created their own private datasets (Ker et al., 2019; Jin et al., 2020), where (Ker et al., 2019) used a full convolutional neural network (CNN) to distinguish normal brain, LGG, and HGG.

#### 2.2.2 Glioma Multiple Classification

Jin et al. demonstrated that a more refined glioma subtype classification would benefit the design of treatment plans (Jin et al., 2020). They developed a new weighted cross-entropy based DenseNet model to automatically classify five types of glioma subtypes: oligodendroglioma, anaplastic oligodendroglioma, astrocytoma, anaplastic astrocytoma, and glioblastoma, with a patient-level accuracy of 87.5%. Nevertheless, the private dataset lacks third-party verification, and another problem is that publicly reproducible studies may not be possible.

The traditional glioma subtype classification under the single modality has insufficient information. Unlike previous studies, automatic classification methods based on multimodal brain images have been recently investigated. These related works mainly came out of the MICCAI 2019 and 2020 CPM-RadPath Challenge (Chan et al., 2019; Pei et al., 2019; Hamidinekoo et al., 2020; Lerousseau et al., 2020; Pei et al., 2020; Yin et al., 2020; Zhao et al., 2020). Based on the CPM-RadPath data, we expect to propose an accurate automatic classification method based on multimodal data.

## 3 Methods

This paper applies two kinds of fully convolutional networks to achieve an end-to-end glioma subtype classification. In the following, we introduce the image preprocessing and network framework in detail.

### 3.1 Data Preprocessing

The MICCAI 2020 CPM-RadPath Challenge provides publicly available H&E stained digital histopathology images and matched multi-sequence radiology images, including three subtypes of gliomas: astrocytoma, glioblastoma, and oligodendroglioma.

Each image in the WSI dataset is very large (e.g., 95,200 × 87,000 pixels) and cannot be directly fed into our network. We crop these WSIs into patches with a size of 1,024 × 1,024 pixels. Then, the OTSU method is adopted to remove non-tissue regions (Otsu, 1979). Following the current studies (Ma and Jia, 2019; Pei et al., 2019; Pei et al., 2020; Pei et al., 2021), we exclude meaningless tissues using a simple but effective threshold technique. Specifically, we first calculate the mean value and standard deviation of each patch in RGB space and maintain patches with a mean value between 100 and 220 and standard deviations above 20 (Pei et al., 2019; Pei et al., 2020; Pei et al., 2021). Then, we convert each patch to the hue saturation value (HSV) space and exclude patches with the mean value below 50 in the H channel (Ma and Jia, 2019).

The volume of the original MRI image is 240 × 240 × 155 pixels. The beginning and end images (slices) in the scan are removed due to their limited brain tissue. As a result, the number of MRI images per sequence is reduced to 128. We also remove the black background at the edges. These MRI images in the four sequences are finally cropped into small images of size 192 × 192 × 128 pixels, which facilitates computational efficiency.

### 3.2 Model Details

**FIGURE 2:**
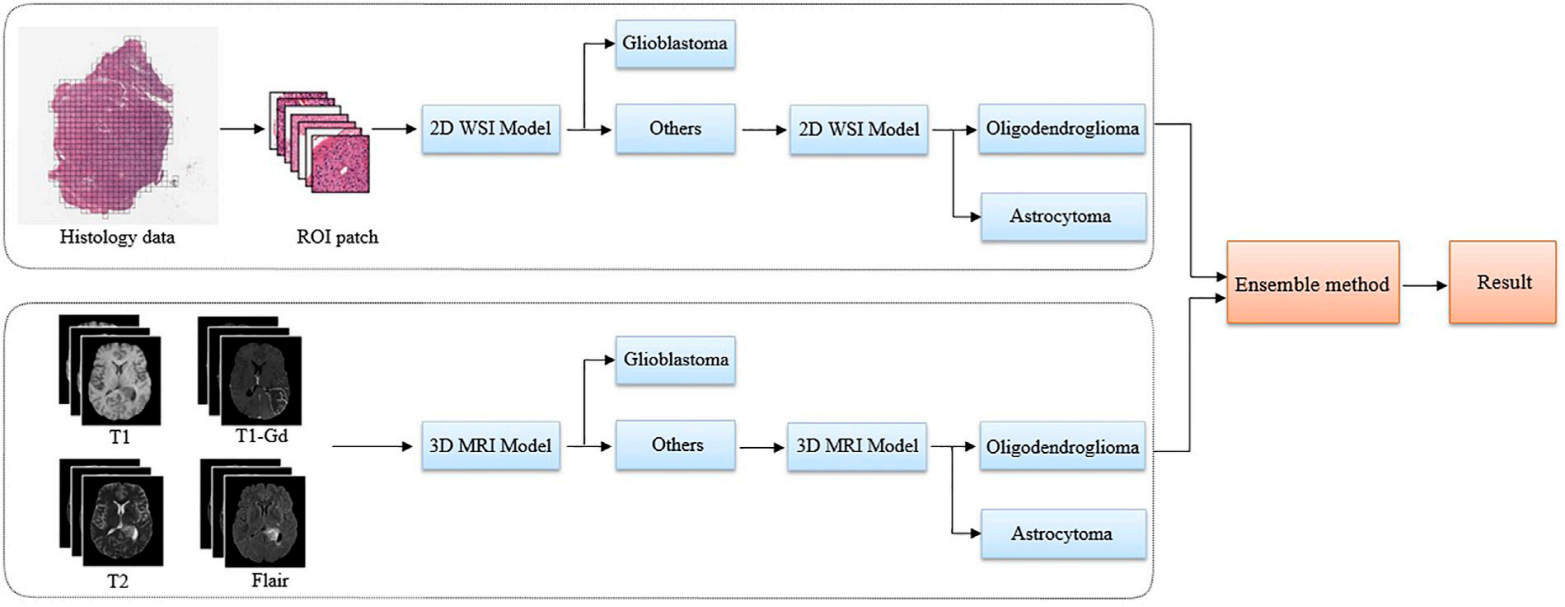
The proposed pipeline using multi-modality data to classify glioma subtypes. A two-stage classification strategy is applied to both the 2D pathology (WSI) and 3D MRI images. The glioblastoma with more serious anatomy representation is detected in the first step. Then, in the second step, our algorithm focuses on the classification of astrocytoma and oligodendroglioma.

**FIGURE 3:**
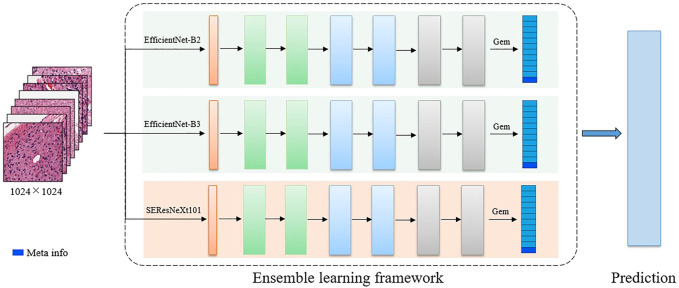
The detailed 2D CNN network. The backbone includes EfficientNet-B2, EfficientNet-B3, and SE-ResNext101. In the final feature representation, the meta-information (age) is included.

**FIGURE 4:**
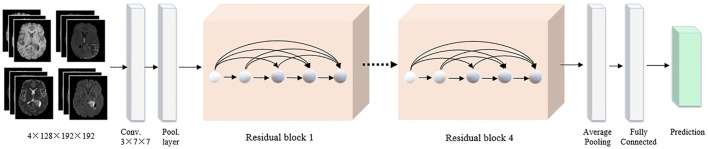
The detailed 3D CNN network. The four MRI modalities are integrated as the network input. All images are cropped to a fixed size of 128 × 192 × 192 pixels. The backbone adopts the 3D ResNet, followed by a global average pooling and a fully connected layer to classify the brain tumor.

Figure 2 illustrates the overall architecture of our proposed glioma classification system, which is composed of a 2D WSI model (Figure 3) and a 3D MRI model (Figure 4). Both models adopt our two-stage strategy. The first stage classifies MRI images or histopathological images as glioblastoma and non-glioblastoma. Based on the obtained non-glioblastoma data, the second stage aims to distinguish astrocytoma and oligodendroglioma. The reason for using the two-stage strategy is the uneven data distribution. The differences between astrocytoma and oligodendroglioma are so subtle that it can be difficult to distinguish between the two. In contrast, glioblastoma is easier to identify. As confirmed by our experimental results, our two-stage strategy helps to improve the overall accuracy.

Multiple related tasks can help learn from each other by potentially sharing representations, thus improving generalization ability. Therefore, for the 2D WSI model, we develop several multi-task-based convolutional neural network models, where the backbones include EfficientNet-B2, EfficientNet-B3 (Tan and Le, 2019), and SEResNeXt101 (Hu et al., 2018). Specifically, EfficientNet is a benchmark network that achieves performance gains by scaling network width, network depth, and resolution, which greatly reduces the number of parameters and computation complexity of the model. EfficientNet-B1 to B7 are obtained by synthetically optimizing the width, depth, and resolution of the EfficientNet. SEResNeXt101 is derived by embedding squeeze-and-excitation (SE) blocks into the ResNeXt model. ResNeXt replaces the original residual learning block (He et al., 2016) with parallel blocks of the same topology, which improves the model performance without significantly increasing the parameters. The SE block obtains the weight of each feature channel and assigns more weights to important features while suppressing features that are not useful for the current task. The outputs of these three classification models are averaged to obtain the final classification results.

Before the fully connected classification layer, the generalized-mean (GEM) pooling (Radenović et al., 2018) is applied to the learned features, which is defined as

$$f^{\left(g\right)}={\left[f_{1}^{\left(g\right)}\ldots f_{k}^{\left(g\right)}\ldots f_{K}^{\left(g\right)}\right]}^{T},\;where,\;f_{k}^{\left(g\right)}={\left(\frac{1}{\left|X_{k}\right|}\sum_{x\in X_{k}}x^{p_{k}}\right)}^{\frac{1}{p_{k}}}$$ (1)

where X is the input features and $f^{\left(g\right)}$ represents the output vector, respectively. $p_{k}$ ∈ [1, +∞), when $p_{k}$ = 1, the equation denotes the average pooling, when $p_{k}$ tends to positive infinity, the equation represents the max pooling, respectively. K denotes the channel of the output feature vector $f^{\left(g\right)}$ . Following (Radenović et al., 2018), this work set $p_{k}$ to 3.

After the GEM operation, age information of patient should also be taken into account (Reni et al., 2017; Ostrom et al., 2018). The reason is that glioma subtypes have different age distributions, where astrocytoma is more often seen in young men while glioblastoma is more frequently distributed in the older groups (Tamimi and Juweid, 2017). In our work, age is used as additional feature, which is concatenated with the extracted image features for the final classification task. Finally, these combined features are adopted as input to a classification branch and a regression branch. The two branches adopt a cross-entropy (L_BCE_) and a smooth L1 loss (L_loc_), respectively, to achieve a more robust brain tumor classification.

$$L_{BCE}=-\underset{l}{\sum }\left[\left(y_{l}\log\hat{y_{l}}\right)+\left(1-y_{l}\right)\log\left(1-\hat{y_{l}}\right)\right]$$ (2)

$$smooth_{L_{1}}=\{\begin{array}{c} 0.5{\left(\hat{y_{l}}-y_{l}\right)}^{2}\;\;\;\;\;\;\;\;\;\;if\;\left|\hat{y_{l}}-y_{l}\right|<1,\\ \left|\hat{y_{l}}-y_{l}\right|-0.5\;\;\;\;\;\;\;\;\;\;\;\;\;\;\;\;\;otherwise.\; \end{array}$$ (3)

$$\;L_{loc}=\underset{l}{\sum }\left[smooth_{L_{1}}\left(\hat{y_{l}}-y_{l}\right)\right]$$ (4)

where $y_{l}$ and $\hat{y_{l}}$ denote the ground-truth and predicted labels for the *l* ^*th*^ sample, respectively.

For the 3D MRI model, the input images are four types of MRI sequence images, including T1, T1-Gd, T2, and FLAIR. Each 3D MRI image is cropped to 192 × 192 × 128 pixels. 3D ResNet is adopted as a backbone to learn the residual representation between the input and output, which has become the basic feature extraction network in the computer vision community. Then, global average pooling and fully connected layers are used to generate the final classification results. We also employ the cross-entropy and smooth L1 loss functions. Finally, the output probabilities of the 2D WSI and 3D MRI models are averaged to drive the final classification results.

## 4 Experimental Results and Discussions

In this section, we first present the utilized data from the MICCAI 2020 CPM-RadPath Challenge and describe the evaluation metrics and experimental setups. Then, we show the detailed classification results and validate the benefits of our proposed multimodal framework and two-stage strategy for glioma classification. Finally, we show the visualization results of three different glioma subtypes in the classification process.

### 4.1 Datasets

We utilize a public dataset released from the MICCAI 2020 CPM-RadPath Challenge^1^, which is proposed for three subtypes of glioma classification, including Glioblastoma, Oligodendroglioma, and Astrocytoma. It includes paired radiology scans and digitized histopathology images with global image-level labels. The CPM-RadPath Challenge splits these data into three parts: training (270 cases), validation (35 cases), and testing (73 cases).

It is noted that only the annotations of training data are released. Thus, our model is developed depending on the training set. The validation and test sets have not released their labels, which can be regarded as two unseen test sets. All these data are collected using multi-parametric MRI (mp-MRI) and digital pathology scanners at 16 international institutions.

Specifically, all mp-MRI scans are acquired using 1–3T scanners. The MRI scans are provided as the NIFTI files (. nii.gz). Each case includes four sequences: T1, T1-Gd, T2, and FLAIR. All MRI images have been preprocessed, co-registered to the same anatomical template, and interpolated to the same resolution (1 cubic mm) in all three directions. Annotations are generated by board-certified neuroradiologists, neurosurgeons, and neuropathologists with at least 4 years of experience.

Meanwhile, tissue specimens are made from tissues removed from the patient during surgery and then stained with hematoxylin and eosin (H&E), which are scanned at 20× or 40× magnification to generate digital histopathology images called WSIs. The color and intensity of WSIs varied across images due to different acquisition times, image fading, or image acquisition artifacts. All WSIs are stored in tiled tiff format. Sixteen professional neuropathologists with at least 4 years of experience annotate these cases by referring to the 2016 WHO classification scheme.

### 4.2 Evaluation Metrics

The algorithmic performance is evaluated using three metrics, including F1-score, balanced accuracy, and Cohen’s kappa. The F1 score is a weighted average of accuracy and recall, which takes into account both false positives and false negatives. The balanced accuracy score is a more appropriate metric to evaluate data with imbalanced categories, which is defined as the arithmetic mean of the proportion of correct predictions in each category. Cohen’s kappa is used for consistency testing and can also be used to measure classification accuracy. A higher kappa coefficient means that the classifier is more effective.

It is assumed that true positive (TP), false positive (FP), false negative (FN) is the number of true positives, false positives, and false negatives respectively. The three metrics can be computed as follows.

$$Sensitivity=\frac{TP}{TP+FN}$$ (5)

$$Precision=\frac{TP}{TP+FP}$$ (6)

$$F1\_Score=\frac{2*\left(Precision*Sensitivity\right)}{Precision+Sensitivity}$$ (7)

$$Balanced\;Accuracy=\overset{c}{\underset{Class=1}{\sum }}Sensitivity/c$$ (8)

$$Kappa=\frac{p_{0}-p_{e}}{1-p_{e}}$$ (9)

In the balanced accuracy score, c is the number of classes. In Cohen’s kappa, $p_{0}$ is obtained by dividing the sum of the number of correctly classified samples by the total number of samples. It is assumed that the true number of samples for each category is $a_{1}$ , $a_{2}$ ,..., $a_{c}$ , and the predicted number of samples for each category is $b_{1}$ , $b_{2}$ ,..., $b_{c}$ . The total number of samples is denoted by *n*. In Kappa, the $p_{e}$ is calculated by

$$p_{e}=\frac{a_{1}\times b_{1}+a_{2}\times b_{2}+\ldots +a_{c}\times b_{c}}{n\times n}$$ (10)

### 4.3 Experimental Setups

Our training data are augmented by horizontal flipping, vertical flipping, random scaling and rotation, and random jitter. We use the ImageNet pre-trained weights to initialize the 2D WSI and 3D MRI models, and the weights of the decoder part are initialized randomly. We used the Adam optimizer (Kingma and Ba, 2015) with an initial learning rate of 3 × 10^−4^ for all experiments. The learning rate decreases by 10 times at the 50^th^ and 80^th^ epochs. The training batch size is set to 24. All networks are implemented based on the PyTorch framework and trained using four NVIDIA Tesla P40 GPU cards.

We used 5-fold cross-validation based only on the training set to find the optimal network parameters for each deep learning model. The best-performing fold is taken as the final training model for the given architecture. It is noted that multiple models with different backbones are trained at each stage of the 2D WSI model, and their ensemble is used to obtain the final results.

### 4.4 The Classification Results

Table 1 shows the results of our ablation experiments conducted on the validation data in the CPM-RadPath challenge. Table 2 shows the contribution of different components in our 2D classification framework. These networks are trained using the same parameter settings as described in the previous section.

**TABLE 1:** Ablation experiment results on CPM-RadPath 2020 validation data.

Method	Balanced accuracy	Kappa	F1 score
3D MRI model (One stage)	0.700	0.665	0.800
3D MRI model (Two stage)	0.733	0.712	0.829
2D WSI model (One stage)	0.767	0.758	0.857
2D WSI model (Two stage)	0.822	0.808	0.886
Ensemble (One stage)	0.800	0.799	0.886
Ensemble (Two stage)	**0.889**	**0.903**	**0.943**

The benefits of the multimodal and two-stage framework for glioma classification efforts. The bold values in the table represent the maximum value of each column.

**TABLE 2:** Experimental performance of the 2D WSI model for ablation on validation data.

Method	Balanced accuracy	Kappa	F1 score
2D WSI model (cls)	0.722	0.659	0.800
2D WSI model (reg)	0.744	0.753	0.857
2D WSI model (cls + reg)	0.800	0.803	0.885
2D WSI model (cls + reg + gem)	**0.822**	**0.808**	**0.886**

“Cls” means a classification branch, “reg” means a regression branch, and “gem” means a fully connected layer. The bold values in the table represent the maximum value of each column.

As the performances shown in Table 1, our two-stage classification strategy contributes to gaining higher accuracy on three evaluation metrics. Moreover, the classification model of MRI and WSI data can complement each other to obtain more robust and accurate results. We conducted a corresponding ablation study to verify the benefits of our proposed multimodal framework and two-stage strategy. We use the same training schedule and parameter settings as the full method described in Section 4.3 for comparison with the ablation method (i.e., with the corresponding components removed).

The experiments demonstrate that our proposed multimodal framework can help improve the accuracy of glioma subtype classification. To validate the importance of multimodal image information, we use MRI or WSI alone as the training set under the most ideal conditions (two-stage), and the balanced accuracy is reduced by 6.7% or even 15.6%. It is well known that in such tasks with unbalanced data, the balanced accuracy provides a better measure of the performance of the algorithm (Brodersen et al., 2010). In addition, there is also a greater than 5% drop in F1 score in this case and a greater than 10% drop in Kappa. In contrast, the multimodal complementary model can reach the best results with 88.9% of the balanced accuracy on the validation set.

Also, we demonstrate the benefit of our two-stage scheme. Another set of experiments is conducted in this study by comparing our two-stage approach with the single-stage alternative. The first stage network is used directly to classify the three glioma subtypes. As shown in the corresponding rows of Table 1, compared to the two-stage approach, the single-stage strategy results in a 3.3 and 5.5% performance decrease in the balanced accuracy in the MRI validation set and WSI validation set, respectively. This result further demonstrates the advantages of our proposed two-stage solution. From the results, it can be seen that our network can integrate feature information from MRI and WSI data.

In addition, we also validate the advantages of the multi-task learning strategy in the 2D WSI model. We use three highly correlated classification networks to train the 2D WSI model: EfficientNet-B2, EfficientNet-B3, and SEResNeXt101. As shown in Table 2, with both classification branch (cls), regression branch (reg), and a fully connected layer (gem), we obtain better results than other methods.

### 4.5 Comparison With the Other Top-Performing Methods in the CPM-RadPath Challenge

Table 3 lists the results of the top six teams in the MICCAI 2020 CPM-RadPath Challenge. As shown in Table 3, our method has obtained the best classification performance in the testing phase.

**TABLE 3:** MICCAI 2020 CPM-RadPath final scores and ranking in the test set.

Rank	Balanced accuracy	Kappa	F Score
Sen (our)	**0.750**	**0.601**	**0.753**
Tabulo	0.662	0.546	0.726
Plmoer	0.654	0.505	0.712
Marvinler	0.652	0.471	0.671
Hanchu	0.519	0.249	0.507
Azh2	0.507	0.209	0.438

Scores in the table are obtained from docker container runs. The bold values in the table represent the maximum value of each column.

In particular, we outperform the second-place team, Tabulo, by 8.8% in terms of balanced accuracy. According to a report submitted to the challenge organizer, Tabulo utilizes two additional publicly available datasets: the Multimodal Brain Tumor Segmentation Challenge 2019 (BraTS-2019)^2^ and MoNuSAC^3^. While our method does not use any external data, all models on the classification pipeline are not pre-trained on medical data. Plmoer, a team from the University of Pittsburgh Medical Center, which processes noisy labels in many patches from WSI of each category. It is similar to us while our two-stage strategy brings an obvious improvement. The Marvinler team used multi-instance learning for the WSI processing, which tends to have high memory requirements. In addition, the Hanchu team requires an additional image segmentation task, yet this does not improve the model performance. The Azh2 team trains a densely connected network for a specific configuration of the DenseNet, which also performs much worse than our model.

It should be noted that we are not able to upload the 3D MRI model in time, so this result is only available for the 2D WSI model with the two-stage strategy. However, it can be seen from Table 1 that the 2D WSI model combined with the 3D MRI model will improve the result substantially. So, we believe that better results will be obtained if the ensemble model is uploaded. Since the test dataset is not yet open, we would like to include the results of the ensemble model with the two-stage strategy if the test dataset is open.

### 4.6 Comparison With Related Works

Table 4 summarizes relevant work on the MICCAI 2019 and 2020 CPM-RadPath Challenge. These results are all obtained on the validation set and are all in the one-stage classification framework.

**TABLE 4:** Comparison with related works on CPM-RadPath validation data.

Studies	Methods	Data	Balanced accuracy	Kappa	F1 score
Pei et al. (Pei et al., 2019)	U-Net model for segment tumors, and 3D CNN model for classification	CPM-RadPath 2019 data set	0.749	0.715	0.829
Chan et al. (Chan et al., 2019)	VGG16 model and Resnet50 model for image feature extraction, and k-means clustering model for classification	CPM-RadPath 2019 data set	—	—	0.780
Hamidinekoo et al. (Hamidinekoo et al., 2020)	DCN model for classification	CPM-RadPath 2020 data set	0.723	0.554	0.714
Yin et al. (Yin et al., 2020)	After the cell kernel segmentation and noise reduction process, 3D Densenet model used for classification	CPM-RadPath 2020 data set	0.944	0.971	0.952
Lerousseau et al. (Lerousseau et al., 2020)	3D Densenet for MRI, and EfficientNet-B0 for WSI	CPM-RadPath 2020 data set	0.911	0.904	0.943
Pei et al. (Pei et al., 2020)	3D CNN for segmentation and classification of MRI, and 2D CNN model for WSI classification	CPM-RadPath 2020 data set	0.800	0.801	0.886
Zhao et al. (Zhao et al., 2020)	VGG16 model for WSI, and segmentation-free self-supervised feature extraction model for MRI	CPM-RadPath 2020 data set	0.889	0.903	0.943
Ours	The two-stage multimodal model for classification	CPM-RadPath 2020 data set	**0.889**	**0.903**	**0.943**

Scores in the table are all obtained from validation set. The bold values in the table represent the maximum value of each column.

The early work (Chan et al., 2019; Pei et al., 2019) first segmented the tumors before classifying them in 2019, however, the final classification results are not satisfactory. In the MICCAI 2020 CPM-RadPath Challenge, (Pei et al., 2020; Yin et al., 2020; Zhao et al., 2020), still use the segmentation before the classification framework, with a significant improvement over last year’s results. The performance of these methods is affected by the segmentation results. Methods in (Hamidinekoo et al., 2020; Lerousseau et al., 2020) and our solutions do not require the segmentation process.

Without segmenting the images, a direct comparison between our method and (Lerousseau et al., 2020) is feasible because of the similar performance on the validation set. However, in the MICCAI 2020 CPM-RadPath Challenge, method in (Lerousseau et al., 2020) performs not well on the test set and the model does not have strong generalization ability. Similarly, although methods in (Yin et al., 2020; Zhao et al., 2020) perform equal or better than our method on the validation set, our method obtains first place on the final competition test set.

### 4.7 Visualization of Results

**FIGURE 5:**
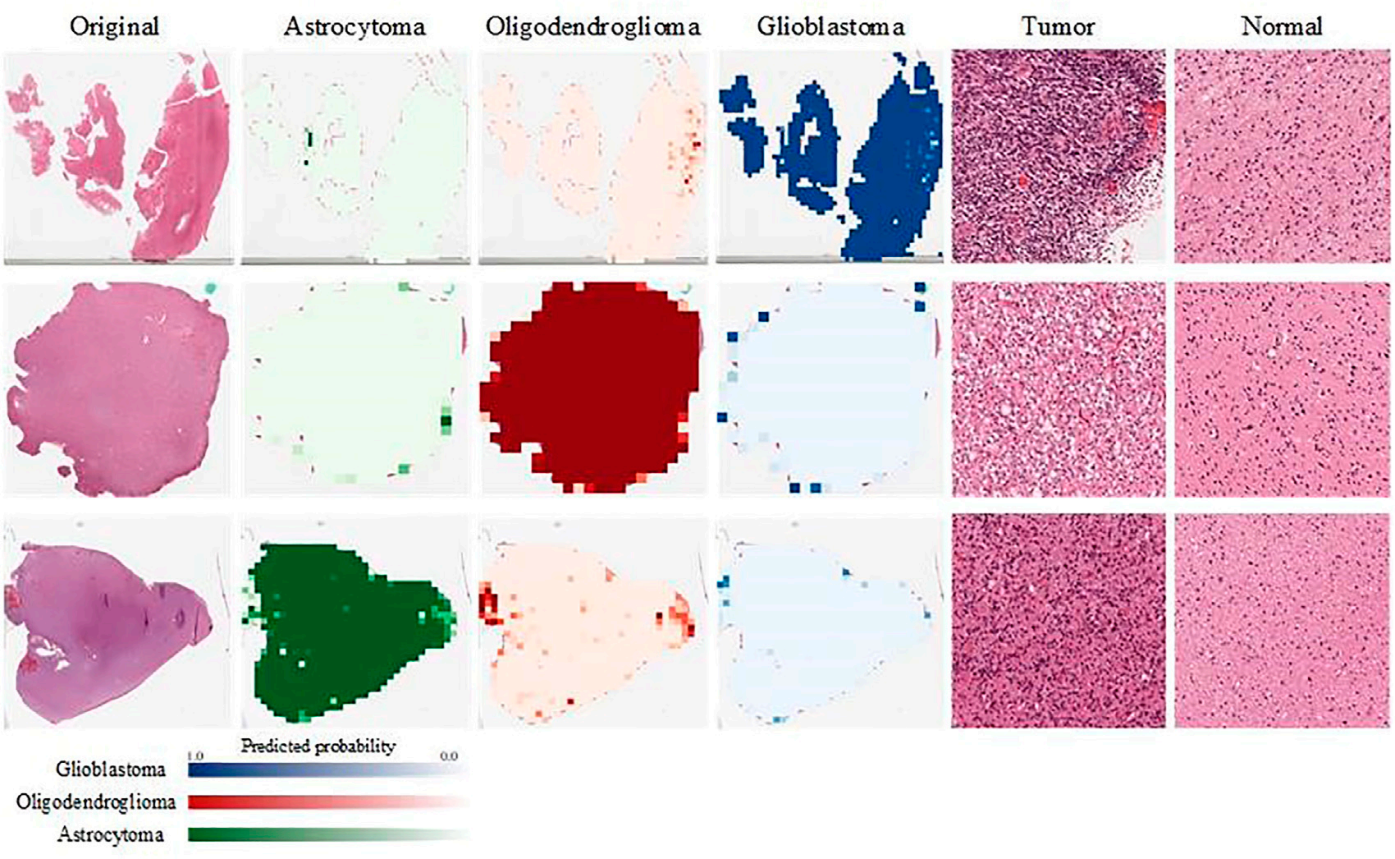
Visualization of the probabilities of the output results. We evaluate the probability that each patch belongs to A/O/G. Green represents A, red represents O, and blue represents G. In addition, we show the patches of different glioma subtypes and normal tissues separately.

We visualize the output of our classification model. We show here the visualization of pathological images of three different glioma subtypes on a classification model. The input patch size is 1,024 × 1,024 pixels, and then each patch is put into our model to calculate the predicted probability for each glioma subtype. Each patch is labeled with the color that represents its probability. Finally, every patch is integrated to obtain the overall probability of the WSI belonging to glioblastoma or oligodendroglioma, or astrocytoma. As shown in Figure 5, probabilities are converted to color maps in logarithmic form. Therefore, those lower probabilities (<50%) are shown as very light colors, and higher probabilities (≥50%) are shown as dark colors.

Two pathologists were invited to view these pathological images and their corresponding patches, and they confirmed the presence of the typical glioma subtype patches and normal patches, which are shown in the right two columns of Figure 5.

## 5 Conclusion

In this paper, we propose a two-stage glioma classification algorithm that integrates multimodal image information to classify brain glioma into three subtypes: astrocytoma, glioblastoma, and oligodendroglioma. We train a 2D WSI model and a 3D MRI model to learn histopathological image information and radiological image information, respectively. Our classification algorithm is designed based on the feature difference between lower and severe glioma grades. In our two-stage strategy, the first stage separates out the more severe glioblastoma, and the second stage focuses only on learning the difference between astrocytoma and oligodendroglioma. Our two-stage strategy is applied to the 2D WSI model and the 3D MRI model, respectively. The ablation experiments show that our proposed multimodal framework and two-stage strategy have achieved more accurate classification performance compared to the unimodal approach and one-stage classification approach. In addition, the 2D WSI model employs an ensemble strategy, which shows higher classification accuracy compared to directly training a single backbone.

Our method has been validated in the publicly available MICCAI 2020 CPM-RadPath Challenge and has ranked first in the challenge, which indicates that the proposed method has the potential to help neurologists or physicians make a fast and accurate glioma diagnosis. However, the limited data is a drawback of this work. Collecting paired multimodal imaging data is difficult due to patient privacy concerns and the heavy clinical workload of physicians. In future work, we will continue to focus on the disclosure of such multimodal data and perform algorithm validation on more data. Further, we will attempt to adopt unsupervised or self-supervised learning techniques to reduce the tedious annotation workload of pathologists.

## Data Availability

The original contributions presented in the study are included in the article/Supplementary Material, further inquiries can be directed to the corresponding author.

## Footnotes

1
https://miccai.westus2.cloudapp.azure.com/competitions/1.

2
https://www.med.upenn.edu/cbica/brats2019/data.html.

3
https://academictorrents.com/details/c87688437fb416f66eecbd8c419aba00dd12997f.
